# Alcohol Vapor Sensor Based on Quasi-2D Nb_2_O_5_ Derived from Oxidized Nb_2_CT_z_ MXenes

**DOI:** 10.3390/s24010038

**Published:** 2023-12-20

**Authors:** Hanna Pazniak, Ilya A. Plugin, Polina M. Sheverdyaeva, Laetitia Rapenne, Alexey S. Varezhnikov, Antonio Agresti, Sara Pescetelli, Paolo Moras, Konstantin B. Kostin, Alexander V. Gorokhovsky, Thierry Ouisse, Victor V. Sysoev

**Affiliations:** 1 Laboratoire des Matériaux et du Génie Physique, Institut Polytechnique de Grenoble, Centre National de la Recherche Scientifique, Université Grenoble Alpes, CS 50257, 38016 Grenoble, Cedex 1, France; laetitia.rapenne@grenoble-inp.fr (L.R.); thierry.ouisse@grenoble-inp.fr (T.O.); 2 Physico-Technical Institute, Yuri Gagarin State Technical University of Saratov, ul. Polytechnicheskaya 77, Saratov 410054, Russia; ilyaplygin@mail.ru (I.A.P.); alexspb88@mail.ru (A.S.V.); kbkostin@gmail.com (K.B.K.); algo54@mail.ru (A.V.G.); 3 Istituto di Struttura della Materia-CNR (ISM-CNR), SS 14, Km 163.5, 34149 Trieste, Italy; polina.sheverdyaeva@ism.cnr.it (P.M.S.); paolo.moras@trieste.ism.cnr.it (P.M.); 4 Center for Hybrid and Organic Solar Energy, Electronic Engineering Department, University of Rome Tor Vergata, 00133 Rome, Italy; antonio.agresti@uniroma2.it (A.A.); pescetel@uniroma2.it (S.P.)

**Keywords:** MXene, gas sensor, niobium oxide, X-ray photoelectron spectroscopy, multisensor array

## Abstract

MXenes are two-dimensional (2D) materials with a great potential for sensor applications due to their high aspect ratio and fully functionalized surface that can be tuned for specific gas adsorption. Here, we demonstrate that the Nb_2_CT_z_-based sensor exhibits high performance towards alcohol vapors at temperatures up to 300–350 °C, with the best sensitivity towards ethanol. We attribute the observed remarkable chemiresistive effect of this material to the formation of quasi-2D Nb_2_O_5_ sheets as the result of the oxidation of Nb-based MXenes. These findings are supported by synchrotron X-ray photoelectron spectroscopy studies together with X-ray diffraction and electron microscopy observations. For analyte selectivity, we employ a multisensor approach where the gas recognition is achieved by linear discriminant analysis of the vector response of the on-chip sensor array. The reported protocol demonstrates that MXene layers are efficient precursors for the derivation of 2D oxide architectures, which are suitable for developing gas sensors and sensor arrays.

## 1. Introduction

Air quality is one of the most important issues in both environmental monitoring and the healthcare industry. Safety rules and standards in these fields are constantly modified to reduce the allowable concentration of toxic gases [1]. Ethanol vapors, which are produced in large amounts in distillation, winemaking and chemical industries, are among these harmful gases. Inhalation of ethanol vapors can cause serious health problems, while their storage at high concentrations can lead to dangerous accidents [2]. To ensure safe working and environmental conditions, gas sensors based on wide-band-gap metal oxide semiconductors like NiO [3], ZnO [4], SnO_2_ [5], and WO_3_ [6] have been developed and employed to monitor ethanol concentration. The working principle of such sensors exploits the change of charge carrier concentration and resistance of the sensing material upon adsorption of gas molecules, known as a chemiresistive effect. However, the ability of pristine oxides to adsorb gases is moderate and must be improved to enhance sensor performance [7]. Moreover, many sensors based on semiconductor oxides use bulk structures that have rather low sensitivity. For this scope, current research in many laboratories deals with (i) doping strategies, (ii) designing heterostructures of two or more materials and/or (iii) enhancing the specific surface area of sensor materials via nano-structuring by growing nanorods, nanowires or spheres, for instance [8].

In this regard, two-dimensional (2D) materials show great promise for sensing applications due to their high specific surface area, which offers a large number of active sites for gas adsorption [9]. Among the expanded family of 2D materials, MXenes are of particular interest due to their tunable terminations and related functional properties. MXenes are early transition metal (M) carbides or nitrides (X), which are derived by selective chemical etching of the A layer (typically Al) from the bulk MAX phase precursor. As a result, MXene surfaces become fully functionalized with abundant groups inherited from the etchants. These groups are usually mixtures of =O, –OH and –F when MXenes are produced by wet chemistry [10] or mixtures of halogen (–Cl, –Br) and =O when molten salts are used as an etchant [11]. Since their discovery in 2011 [12], MXenes have demonstrated excellent performance in gas-sensing applications. In particular, Ti_3_C_2_T_x_, V_2_CT_x_ and Mo_2_CT_x_ are reported to be good for the selective detection of ammonia [13,14], nonpolar gases [15] and water vapors [16] at low concentrations. In addition, due to the hydrophilicity of MXenes, solution processed deposition methods can be easily adapted for the fabrication of gas sensors.

Over the last few years, various MXene chemistries have been synthesized and investigated from a fundamental and applicative point of view [17,18]. Nb_2_CT_z_ is one of the promising phases whose properties drastically depend on the nature of the surface functional groups [19]. Nb_2_CT_z_ has already been tested for applications in energy storage [20] and biomedicine [21], but limited information is available on the gas sensing properties of this phase. There are only a few studies demonstrating the sensitivity of Nb_2_CT_z_-based optics fiber sensors for tyramine [22] and humidity [23] detection or reporting the performances of Nb_2_CT_z_-based chemiresistive sensors for NO_2_ [24] and NH_3_ [25] vapors. The enhanced sensitivity of these sensors was achieved either by functionalization of Nb_2_CT_z_ with amino groups [24] and Au nanoparticles [22], or by mixing Nb_2_CT_z_ with nano- [25] or microfibers [23].

Herein, we explore the gas-sensing properties of pristine Nb_2_CT_z_ MXenes with respect to various volatiles, including alcohols, ammonia and water vapors. We show that sensors based on pristine Nb_2_CT_z_ exhibit no response at room temperature. Instead, when the operating temperature of the sensor is increased, reliable sensor performance is obtained at 300–350 °C. We ascribe this behavior to the oxidation of MXenes to the 2D Nb_2_O_5_ oxide phase as directly evidenced by X-ray photoelectron spectroscopy (XPS) and X-ray diffraction (XRD). These results are in line with our previous research where a substantial improvement of chemiresistive properties of pristine MXenes is attained via their oxidation to form 2D oxide heterostructures [26].

## 2. Materials and Methods

### 2.1. Synthesis and Characterization of Nb_2_CT_z_ MXenes

Nb_2_CT_z_ MXenes were synthesized via a selective chemical etching of Al layers from a bulk Nb_2_AlC MAX phase precursor in a one-step procedure using a mixture of LiF and HCl according to the protocol reported by J. Xiao et al. [27]. In detail, 2.0 g of LiF was slowly added to 20 mL of 12 M HCl solution and left under constant stirring for 5 min. The vial with the solution was placed in an ice bath where Nb_2_AlC powder was carefully added in minor portions. After that, the mixture was kept for 90 h at 60 °C under continuous stirring. Following the reaction, the solution was washed multiple times with deionized water, with further centrifugation at 3500 rpm for 5 min until a nearly neutral pH value was obtained. The presence of Li ions in the etching solution allows for a spontaneous Li intercalation between the MXene layers during the etching. This effect favors further delamination of multilayers into single or few-layered sheets using a vortex shaking process. After the vortex shaking, the mixture was centrifuged again until a stable suspension of well-delaminated Nb_2_CT_z_ sheets appears. This step was repeated several times, and the supernatant was collected over the PVDF membrane through vacuum-assisted filtration to be dried under vacuum, with residual pressure down to approx. 30 mbar, at room temperature (RT).

Scanning electron microscopy (SEM) of the Nb_2_AlC MAX phase and Nb_2_CT_z_ MXenes was performed using a FEG ZEISS GeminiSEM 300 instrument (Carl Zeiss AG, Oberkochen, Germany). SEM images of the sensor chip were recorded using an Aspex Explorer microscope (FEI, Delmont, PA, USA) equipped with an energy dispersion X-ray (EDX) detector. X-ray diffraction (XRD) patterns were recorded using a D8 Bruker diffractometer (Bruker, Billerica, MA, USA) operating under the Bragg–Brentano geometry and equipped with a Cu anode providing X-rays with a 1.5418 Å wavelength. Transmission electron microscopy (TEM) images were acquired with an JEOL 2010 microscope (JEOL, Akishima, Japan) at an acceleration voltage of 200 kV. High-resolution X-ray photoelectron spectroscopy (HR-XPS) measurements were performed in the VUV-Photoemission beamline of the Elettra synchrotron facility (Trieste, Italy).

In order to evaluate the thickness of the Nb_2_CT_z_ layer coated over the chip, we applied a stylus profilometry (stylus profiler Dektak 150, Veeco, Plainview, NY, USA) with a stylus of 2 μm radius under a force equal to 1 mg. The resolution of measuring points was approx. 0.05 μm.

### 2.2. Gas-Sensing Experiment

To perform gas sensor measurements, we employed the multielectrode chip platform [28]. In brief, the chip is a Si/SiO_2_ substrate patterned with Pt strip electrodes with 50 µm gaps [29]. To deposit the MXene layer on the chip, a dispersion of delaminated Nb_2_CT_z_ in isopropanol, 0.1 mg/mL, was sprayed onto a working area of the chip, of 0.215 mm^2^, using a spray brush system. The distance between the nozzle tip of the airbrush and the multielectrode chip was adjusted to 7 cm. The flow rate of the MXene suspension was optimized to 5 s/drop by changing the position of the airbrush needle. The deposition pressure was adjusted to 1 bar and the deposition temperature to 100 °C. The thickness of the Nb_2_CT_z_ layer was controlled by the number of deposition cycles.

**Figure 1:**
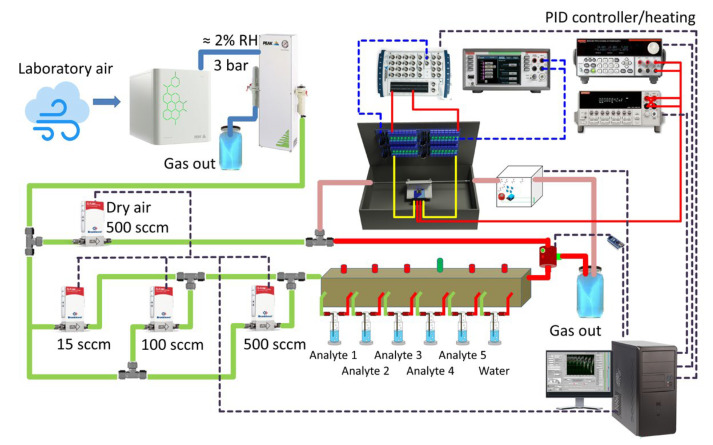
The experimental setup to measure the response of the Nb2CTz MXene-based chip to various analyte gases emitted via bubbling. The tube lines containing a background air and analyte-enriched air are marked by light green and red, correspondingly.

After the preparation, the chip was connected with Al wires, 38 μm diameter, to a window in the ceramic holder equipped with multiple metallic thin-film roads to contact pads and 50-pin interface (Erni SMC, 1.27 mm, Erni Electronics GmbH, Adelberg, Germany) for connecting the external reading electronic circuits. For analyte gas supply, the Nb_2_CT_z_-based chip-in-holder was introduced to an alloy steel-fabricated chamber supplied with two sealing heat-tolerant rubber rings. The chamber was blown with a gas flow via in-mounted tubes, 1/8′ in diameter, to feed the probes primarily to the chip frontside. Figure 1 shows the measuring setup for conducting the gas-sensing measurements with the chip under study. The whole chamber and all the wires were put in a grounded metal box in order to avoid any influence of the external electromagnetic field.

All the electrode pairs in the on-chip sensor array were measured upon applying d.c. electric potential, up to 5 V, in sequence via multiplexing relays (Songle Relay, Ningbo, China) to record the current and derive the resistance of each layer area between electrodes. These resistances, up to 10 GOhm, were processed as sensors of the chemiresistive type. The current was recorded by a multimeter (Keithley DAQ6510, Keithley Instruments, Solon, OH, USA) to be fed via an analog I/O unit (USB-6259, National Instruments Co., Austin, TX, USA) with a rate of up to 300 ms per sensor. Two modes of electrical readings were employed in the course of the study: (i) measuring I(V) dependencies while applying the electric bias in the range of [−5;+5] V mainly in background air conditions to evaluate the contact between the layer under study and electrodes, and (ii) measuring I(t) transients converted to R(t) ones while applying a constant bias of 5 V to see the resistance changes upon exposing to various gas probes. A PC operated with LabView^@^-based software (LabView Full Development System with Application builder, ver. 2020 SP1, National Instruments Co., Austin, TX, USA) was used for this setup.

The gas-sensing measurements were performed when supplying analyte gases via tubes, 1/4′ diameter, joined by Swagelok fittings (Swagelok Co., Solon, OH, USA) at a flowrate of 400 sccm to be managed by high-precision mass-flow controllers (Bronkhorst, Ruurlo, The Netherlands). Regarding the background air, we employed a lab one to be filtered and dried using a corresponding generator (Purge PG14L, Peak Scientific, Glasgow, UK) purged with a compressor (Precision Compressed Air 230v, Peak Scientific Instruments Ltd., Glasgow, UK) yielding 40 psi pressure to feed the total gas delivery system.

To enrich the air with analyte probes, we used bubblers (Gebr. Rettberg GmbH, Göttingen, Germany) containing liquids of distilled water, various alcohols (methanol, ethanol, isopropanol, butanol) and 25% NH_3_ solution in water. The saturated vapors of each analyte emitted primarily following the bubbling were diluted by air at necessary ratios to yield analyte probes in concentrations of 50–8000 ppm where the limits were managed by the employed flow controllers and physical properties of the analyte liquids. In the case of measurements in humid air as a background, the dry air was first passed through a water-based bubbler to get containing 25% relative humidity or ca. 10,400 ppm H_2_O concentration. The analyte gas exposures to the chamber with the installed chip were monitored independently by a commercial semiconductor sensor (MQ3, Winsen Co., Zhengzhou, China). The analytes of each type/concentration were supplied to the chip for 90 min to be further cleared by pure background air, which was dry or humid depending on the targeted experimental conditions, for 180 min at least. The measurements of the chemiresistive response of the Nb_2_CT_x_ film as a layer in the chip were conducted at a temperature range of 25–300 °C to be maintained by the chip in-built meander heaters. The power dissipated over the heaters was managed by a proportional-integral-derivative (PID) controller accounting for data from local on-chip thermoresistors which were initially calibrated with a high-resolution IR camera (InfRec R500EX-P-D, Nippon Avionics Co., Yokohama, Japan) equipped with a 21 μm close-up lens.

## 3. Results and Discussion

### 3.1. Synthesis and Characterization of Nb_2_CT_z_ MXenes

**Figure 2:**
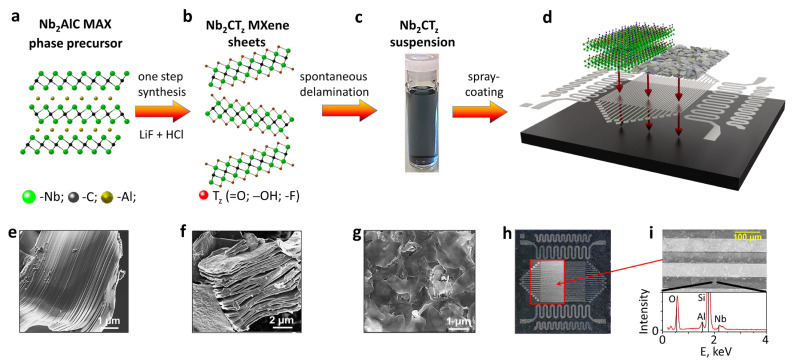
Schematic depiction of Nb2CTz MXene synthesis and the Nb2CTz-based multielectrode chip: (**a**) structure of the Nb2AlC MAX phase precursor; (**b**) structure of Nb2CTz sheets produced via a selective chemical etching of the Al layer in the mixture of LiF and HCl; (**c**) photo of the delaminated suspension of Nb2CTz MXenes; (**d**) cartoon of the chip fabrication; (**e**–**g**) SEM images of MAX precursor (**e**), multilayered MXenes (**f**) and delaminated Nb2CTz sheets in the layer (**g**); optical and SEM images of the chip covered by Nb2CTz layer via a spray casting and EDX spectrum taken in the inter-electrode gap (**h**,**i**).

To produce Nb_2_CT_z_ MXenes, a selective chemical etching of Al layers from bulk Nb_2_AlC was carried out in a one-step procedure using a mixture of LiF and HCl as detailed in the Section 2. These etchants are widely used for the synthesis of Ti_3_C_2_T_x_ MXenes and allow one to produce large flakes under a mild delamination condition. We have adapted this procedure to obtain Nb_2_CT_z_, which is schematically illustrated in Figure 2. The Nb_2_AlC MAX phase consists of alternating Nb_2_C and Al layers (Figure 2a) and exhibits a densely packed structure (Figure 2e) as revealed by SEM. After the selective removal of Al, a characteristic accordion-like multilayer structure appears (Figure 2f) due to the release of H_2_ gas arisen during the reaction of Al and HF. This structure indicates the conversion of the MAX phase to MXenes, which was additionally confirmed by XRD. The synthesis of Nb_2_CT_z_ under optimized conditions is reproducible to yield MXenes with the same structural characteristics.

**Figure 3:**
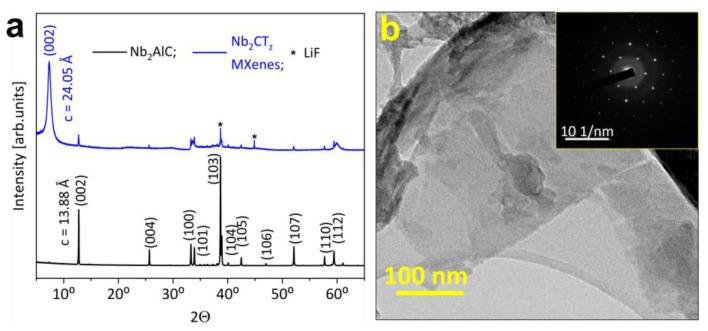
Structural characterization of Nb2CTz MXenes. (**a**) XRD patterns of Nb2AlC MAX phase precursor and derived Nb2CTz MXenes by the chemical etching of Nb2AlC; (**b**) TEM image of Nb2CTz flakes with corresponding SAED pattern (inset).

The XRD plots given in Figure 3a show that the (002) peak of the parent MAX precursor significantly shifted after the MXene synthesis to the lower 2θ position, which corresponds to a change in the c-parameter from 13.88 Å to 24.05 Å. This is due to an increase in the interplanar spacing as a result of Al etching and subsequent intercalation of water and Li ions.

The XRD pattern of the MXenes also reveals the presence of low-intensity peaks of the initial precursor. However, unreacted MAX phase and non-delaminated MXene multilayers are easily separated from the delaminated suspension.

The presence of Li ions in the etching solution favors a further delamination of multilayers into single or few-layer sheets. After delamination, Nb_2_CT_z_ suspension exhibits a characteristic light blue color when diluted (Figure 2c) [30]. We drop-cast the delaminated suspension over the Si/SiO_2_ substrate and observed the poly-flake morphology of the deposited layers using SEM (Figure 2g). It is clearly seen that the film consists of translucent flakes, irregular in shape and of rather large sizes (1–2 µm). From the observation of single MXene flakes in TEM, we notice that Nb_2_CT_z_ exhibits typical flake features, which are illustrated in Figure 3b, similar to delaminated Ti_3_C_2_T_x_ [26]. The selected area electron diffraction (SAED) pattern of the flakes shows (inset, Figure 3b) the hexagonal arrangement of atoms which retains the symmetry of the parent precursor and confirms the high crystallinity of the produced 2D sheets. It is important to note here that as-prepared pristine Nb_2_CT_z_ MXenes do not exhibit any oxidation, as evidenced by the absence of oxide peaks in XRD and SAED patterns.

**Figure 4:**
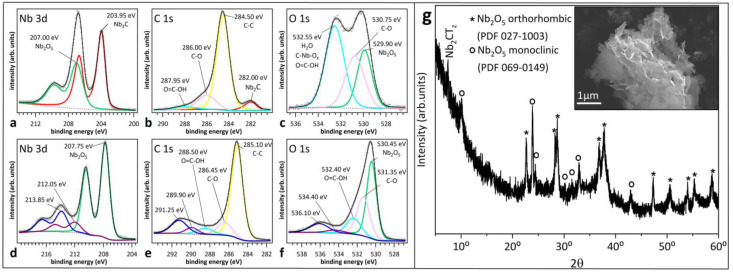
HR-XPS data of as-prepared Nb_2_CT_z_ MXenes (**a**–**c**) and after oxidation at 350 °C (**d**–**f**). XRD pattern and SEM image (inset) for the oxidized Nb_2_CT_z_ MXenes (**g**).

To clarify the physical processes occurring in the Nb_2_CT_z_ MXene layer under heating, we performed high-resolution X-ray photoelectron spectroscopy (HR-XPS) measurements. The data are summarized in Figure 4: top row spectra refer to as-synthesized Nb_2_CT_z_, while bottom row spectra refer to Nb_2_CT_z_ exposed to 350 °C. Figure 4a reveals the presence of two Nb 3d doublets, which correspond to Nb-C in the MXene (Nb 3d_5/2_ at 203.95 eV, metallic lineshape) and to Nb_2_O_5_ (Nb 3d_5/2_ at 207.00 eV) that forms spontaneously during the process of exfoliation. In the C1s spectrum, there are four components (Figure 4b). The peak located at 282.00 eV is the counterpart of the Nb-C doublet and has an asymmetric shape, thus confirming the metallic nature of Nb_2_CT_z_. The other peaks are assigned to adventitious carbon (284.50 eV), C–O (286.00 eV) and O=C–OH (287.95 eV). The O1s spectrum can be described with three peaks (Figure 4c) corresponding to Nb_2_O_5_ (529.9 eV), C–O (530.75 eV) and the convolution of adsorbed water, O-containing termination of MXenes and O=C–OH (532.55 eV).

After exposure of the spray-coated Nb_2_CT_z_ film to 350 °C, there was no signature of metallic Nb-C bonds either in the Nb 3d spectrum (Figure 4d) or in the C 1s spectrum (Figure 4e). This indicates that Nb_2_CT_z_ MXenes apparently tend to oxidize faster than Ti_3_C_2_T_x_ due to the presence of two transition metal layers instead of three ones that are in direct contact with air. The Nb 3d spectrum of Figure 4d consists of three doublets (Nb 3d_5/2_ at 207.75, 212.05 and 213.85 eV) assigned to Nb_2_O_5_ oxide. These shifted doublets originate from charging effects of different magnitudes (0.75, 5.05 and 7.85 eV towards higher binding energy with respect to 3d_5/2_ in Figure 4a) characterizing different parts of the sample. This interpretation is substantiated by the observation of similar shifted components in the C1s and O 1s spectra (Figure 4e,f). The peak of adventitious carbon in Figure 4e is located 0.60 eV deeper than the corresponding peak in Figure 4b. The two features at 289.90 and 291.25 eV are mainly ascribed to adventitious carbon peaks shifted by stronger charging effects. Similarly, the Nb_2_O_5_ peak in the O 1s spectrum of Figure 4c is located 0.55 eV deeper than the corresponding peak in Figure 4f. Also, two additional shifted components are observed (534.40 eV and 536.10 eV) in this case [31]. The XRD pattern confirms the absence of Nb_2_CT_z_ and the coexistence of orthorhombic and monoclinic crystallographic phases of Nb_2_O_5_ oxide. The large and phase-dependent bandgap of Nb_2_O_5_ (3.1–5.3 eV) can explain the different energy shifts of the photoemission peaks observed in Figure 4d–f. Notably, the SEM image in the inset of Figure 4g shows that the surface of the oxidized sample has a thin flake-like structure that differs from the typical morphology of bulk Nb_2_O_5_ particles.

### 3.2. Gas-Sensing Measurements

**Figure 5:**
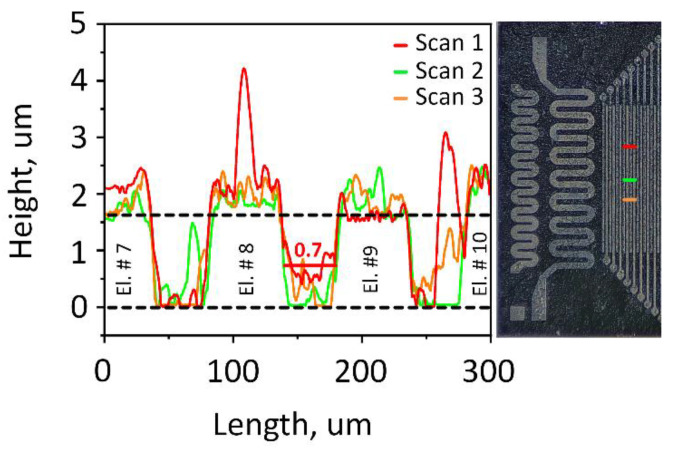
The stylus profile scans taken over the multielectrode chip coated with an oxidized Nb_2_CT_z_ MXene layer following gas-sensor measurements at high temperatures. The spot of four electrodes, #7–10 counting from left to right in the chip, is taken for example.

To perform gas sensor measurements, we employed the multielectrode chip platform as detailed in the Section 2 [28,29]. While several samples were prepared at different stages of research, here we show the data related to exemplary chip to be fully characterized in order to highlight its major features. The deposited Nb_2_CT_z_ sheets (Figure 2h,i) cover the working area of the chip in the form of a percolating layer. The profiles of the layer were taken across the electrodes of the chip. The height of the electrodes is approximately 1.5 µm and is shown by the black dashed line in Figure 5. The Nb_2_CT_z_ layer is lying between and over the top of the electrodes; its height profile is marked in red, orange and green, corresponding to different scans. The layer thickness is estimated as the difference between cross-electrode profiles of the pristine and the coated chip and does not exceed 700 nm in the inter-electrode gaps.

The EDX plot (bottom part of Figure 2h) clearly indicates the presence of Nb from the MXenes, Si and O from the substrate (due to the small thickness of the spray-coated film) and residual traces of Al.

**Figure 6:**
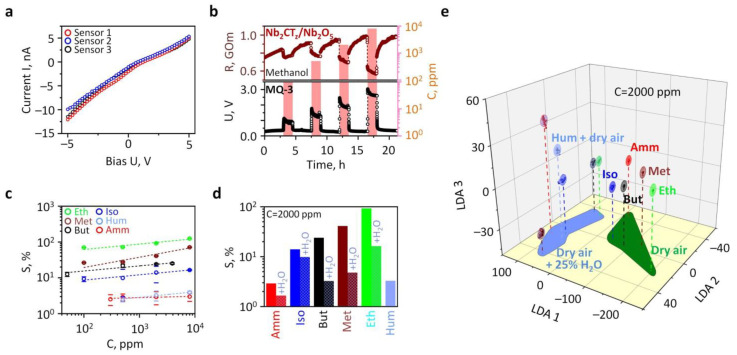
Gas-sensing performance of the Nb_2_CT_z_/Nb_2_O_5_-based chip at 300 °C: (**a**) I-V curves measured in dry air for three sensor elements; (**b**) typical R(t) transient of single sensor element (top) and voltage signal of commercial sensor exposed to methanol vapors; (**c**) the chemiresistive response of the sensor elements toward various analyte concentrations; (**d**) selectivity diagram of chemiresistive response at an analyte concentration equal to 2000 ppm; (**e**) 3D cross-section of the total 10D LDA space; the points relate to chip vector signals to analytes, 2000 ppm concentration, ellipses frame areas for vector data scattered under Gaussian distribution with 0.9 confidence around gravity centers of analyte-related clusters; abbreviations: Amm—ammonia; Iso—isopropanol; But—butanol; Meth—methanol; Eth—ethanol; Hum—humidity.

After the chip preparation and its storage under air conditions, the gas-exposure tests at room temperature revealed no changes in the resistance of the Nb_2_CT_z_ film that went beyond the range of 10 GOhm that could be measured. It would seem that the MXenes were spontaneously oxidized upon deposition, probably in the form of Nb_2_CT_z_/Nb_2_O_5_ or Nb_2_O_5_ phases, resulting in non-measurable conductivities. Therefore, to activate the film conductance, we sequentially heated the chip and could then measure the resistance, still in the GOhm range, at temperatures close to 300 °C. Figure 6a shows I-V curves measured when the chip was exposed to dry air. Although the curves look rather linear, these data indicate the existence of a slight Schottky barrier between the layer and Pt electrode related to differences in the work functions of these materials [32]. When the chip was exposed to test analyte vapors, we could observe a reliable reduction in resistance with a magnitude depending on the analyte concentration. The typical R(t) transient is shown in Figure 6b for the case of methanol vapors mixed with dry air, at a concentration of 100–8000 ppm. For comparison, we show the signal of a commercial semiconductor sensor given as an electric bias, *U*, in volts to the same exposures. As one can see, resistance of the Nb_2_CT_z_ film fully follows the varying signal of the commercial sensor although at a lower amplitude change. Similar behavior was observed when the chip was exposed to other test analytes, which is consistent with the chemiresistive effect in n-type wide-band semiconducting oxides [33], which highlights the reducing nature of the test analytes.

The major sensor characteristic, the calibration function of the response, *S*, versus gas concentration, *C*, is given in Figure 6c in a double log scale. The response is defined as a relative change in film conductance in percent:

(1) $$S=\left(\frac{G_{i}(gas)}{G_{i}(air)}-1\right)\times 100,$$

where $G_{i}(gas)$ and $G_{i}\left(air\right)\;$are the local conductances of the film located between each couple of electrodes (sensor element) recorded upon an exposure to the analyte gas probe and background air, respectively.

As one can see, all the *S*(*C*) curves describing the analytes follow the $S\sim C^{n}$ function to be compatible with the Freundlich adsorption isotherm. The power exponent values related to the analyte probes are listed in Table 1. The lowest value of *n*, ca. 0.04, is observed for ammonia vapors, which yields a very limited sensor response, down to ca. 1–2%, in contrast to alcohol vapors where *n* varies between 0.14 and 0.29; still, the response magnitude to alcohols lies in the range of 8–100%. It is interesting that humidity also exhibits a reduced chemiresistive effect with a magnitude of sensor response similar to ammonia, although the *n* value of 0.16 for this analyte corresponds to those characterizing alcohols.

To further elucidate the effect of H_2_O vapors, we performed studies exposing the chip to the same analytes mixed with wet air, at 25% RH. These data are summarized in Figure 6d along with data observed in dry background air. As one can see, the presence of H_2_O in a background atmosphere substantially lowered the sensor response to all the analytes, although the signals are still in the range of 2–11% at a concentration of 2000 ppm. The observed chemiresistive effect is very similar to one reported earlier with non-stoichiometric Nb_2_O_5_ oxide [32,34], and it matured due to the presence of defects, primary oxygen vacancies and redox surface reactions. Still, it can be assumed that the possible contribution from the layer/electrode barrier is driven by a similar nature, when the contact potential depends on the same processes that occur with the material under study itself. As shown above, the Nb_2_CT_z_ film transforms into an oxide phase at temperatures of 300–350 °C. The latter is known to have a strong non-stoichiometry in oxygen, preferentially observed in high-surface-area nanostructured materials [32]. We assume that the Nb_2_O_5_ formed as a result of the oxidation of Nb_2_CT_z_ is non-stoichiometric, since a well-crystalline oxide is formed at a higher temperature of 800 °C [33]. When a non-stoichiometric Nb_2_O_5_ film is exposed to wet air (or a humid atmosphere), OH-groups are adsorbed on an oxygen lattice defect, which then reduce the sensor signal from analytes. This non-stoichiometry of the derived quasi-2D Nb_2_O_5_ can also explain its higher signal towards alcohols compared to ammonia in accordance with other data reported in [34]. Interestingly, while our previous study showed that Ti_3_C_2_T_z_ MXenes annealed at 350 °C display the highest response towards acetone [26], Nb_2_CT_z_ MXenes annealed at the same temperature showed the highest response towards ethanol. This indicates that the selectivity of MXenes is highly dependent on transition metals and derived oxides.

**Figure 7:**
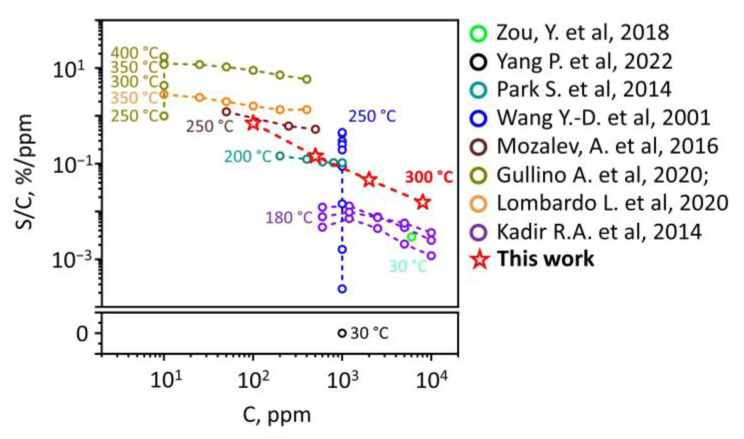
A comparative plot of sensor performance of pristine Nb_2_O_5_ structures versus ethanol vapors based on literature data (adapted from Refs. [35,36,37,38,39,40,41,42]) regarding the results presented here. S/C is the response (S)-to-concentration (C) ratio.

Still, the performance of the derived sensor toward ethanol vapors is comparable or exceeds one reported in the literature for the pristine Nb_2_O_5_ of various morphologies. We have compared these data in Figure 7.

However, we cannot selectively distinguish the analytes by taking the signal of a single sensor element. Therefore, for the selectivity issue, we could consider the vector signals generated by the whole sensor array located on the chip, regarding all the test analytes recorded in stationary parts of R(t) curves [43]. The multisensory vector signal was processed with the linear discriminant analysis (LDA) algorithm using homemade software. This technique reduces the original multidimensional signal of the array, whose dimensionality is equal to the number of sensors, into an artificial space of features with the dimensionality of (*N* − 1), where *N* is the number of classes (here, analytes) to recognize [44,45]. In essence, the LDA processing is performed via maximizing the inter-class variation of vector signals versus their in-class scatter. The vector array data given, for example, in the case of analytes present at a concentration of 2000 ppm, are shown in the LDA space of Figure 6e, where they are grouped according to origin. Here, we display a 2D cross-section of the total 10D LDA coordinate system.

As one can see, the gravity centers of the analyte-related clusters are well distinguished here with an average Mahalonobis distance of 130 units. While the zero point in this space relates to fully noisy data like “white noise” that has no specific features toward the inputs under study, this distance could serve as an indication of selectivity. From the obtained data, it is clear that all the analytes could be distinguished with the developed on-chip array.

## 4. Conclusions

Thus, the present results show that 2D sheets of Nb_2_O_5_, which appeared following the oxidation of Nb_2_CT_z_ MXenes in the temperature range 300–350 °C, could be beneficial for application in gas sensors to detect alcohol vapors, among which the highest response is observed towards ethanol (~100% at 2000 ppm). The combination of various thorough structural characterizations of the material reveals that the improvement in sensing performance is likely due to the flake-like morphology of the derived Nb_2_O_5_ oxide and its non-stoichiometry.

Here, we should note that room-temperature-operated sensors are highly attractive from a power consumption viewpoint. However, in practically all the materials, such an operation appeared mostly in 2D architectonics, which suffers from the long-term impact of adsorbed species. It ordinarily yields a significant shift into the baseline under the high influence of humidity and/or other VOCs and gases present in the normal gas atmosphere. To date, even UV illumination does not give the necessary power to accelerate the rate of adsorption/desorption of gas molecules from the material surface. Therefore, heating remains the “gold standard” for operating chemiresistors as these new data further support.

The reported protocol is easily applied for developing multisensor arrays of the planar type for Enose fabrication with a high sensitivity towards alcohols mixed with dry and wet air in concentrations corresponding to known detection requirements like the regulations by the U.S. Occupational Safety and Health Administration (OSHA). Therefore, it could be considered for various applications including the Internet of Things.

## Figures and Tables

**Table 1:** The power index, *n*, of the *S*(*C*) curve characterizing the gas response of sensor elements of the Nb_2_CT_z_/Nb_2_O_5_-based chip operating at 300 °C versus various test analytes.

**Gas**	Ammonia	Humidity	Isopropanol	Butanol	Methanol	Ethanol
***n***	0.04 ± 0.02	0.16 ± 0.02	0.15 ± 0.03	0.14 ± 0.04	0.29 ± 0.06	0.15 ± 0.03

## Data Availability

Data are contained within the article.
